# The -271 G>A polymorphism of kinase insert domain-containing receptor gene regulates its transcription level in patients with non-small cell lung cancer

**DOI:** 10.1186/1471-2407-9-144

**Published:** 2009-05-12

**Authors:** She-Juan An, Zhi-Hong Chen, Qiu-Xiong Lin, Jian Su, Hua-Jun Chen, Jia-Ying Lin, Yi-Long Wu

**Affiliations:** 1 Medical Research Center of Guangdong General Hospital, Guangdong Lung Cancer Institute, Guangdong Academy of Medical Sciences, Guangzhou 510080, PR China

## Abstract

**Background:**

Kinase insert domain-containing receptor (KDR) plays a critical role in the metastasis of cancer and is used as a molecular target in cancer therapy. We investigated the characteristics of the -271 G>A polymorphism of the KDR gene to gain information that may benefit the development of individualized therapies for patients with non-small cell lung cancer (NSCLC).

**Methods:**

The -271 G>A polymorphism of the KDR gene in 106 lung cancer patients and 203 healthy control individuals was analyzed by polymerase chain reaction (PCR) and DNA sequencing methods. Real-time quantitative PCR and immunohistochemical methods were used to evaluate KDR mRNA and protein expression levels, respectively, in frozen tumor specimens.

**Results:**

The -271 G>A polymorphism was associated with the mRNA expression level of the KDR gene in tumor tissues (t = 2.178, P = 0.032, independent samples *t*-test). Compared with the AG/GG genotype, the AA genotype was associated with higher KDR mRNA expression in tumor tissues. We found no relationship between the genotype and the KDR protein expression level and no significant difference in the distribution of the KDR gene polymorphism genotypes between lung cancer patients and the control group (χ^2 ^= 1.269, P = 0.264, Fisher's exact test).

**Conclusion:**

This study is the first to show that the -271 G>A polymorphism of the KDR gene may be a functional polymorphism related to the regulation of gene transcription. These findings may have important implications for therapies targeting KDR in patients with NSCLC.

## Background

Lung cancer is a leading cause of cancer deaths in the United States and throughout the world, in both men and women [1,2]. The development and progression of lung cancer is a multi-step process, characterized by the accumulation of multiple genetic and epigenetic alterations that perturb regulatory and growth-control pathways in the cell [3,4]. The prognosis is poor, with only 10–15% of patients surviving 5 years after diagnosis, owing to a lack of efficient diagnostic methods for early detection and a lack of successful treatment for metastatic disease.

Angiogenesis is an essential process in the development, growth, and metastasis of malignant tumors, including lung cancer tumors. Currently, a key therapeutic strategy is to inhibit specific processes essential for tumor vascular development. A number of anti-angiogenic agents with anti-angiogenic and anti-tumor activity, including a tyrosine kinase inhibitor (TKI), are currently in development [5-8]. The kinase insert domain-containing receptor (KDR; also known as vascular endothelial growth factor receptor 2: VEGFR-2) gene plays a critical role in cancer metastasis and is used as a molecular target in cancer therapy [9-11]. However, little is known about its polymorphisms and the functional significance of its association with lung cancer.

The genetic variations of KDR may influence its systemic production and its effects on vascular endothelial cells in cancer patients, consequently causing individual differences in the responses of patients treated with therapies targeting KDR. Therefore, we analyzed genetic polymorphisms in the 5' untranslated region (UTR) of the KDR gene in Chinese patients with lung cancer, as well as the relationships of these polymorphisms to KDR mRNA and protein expression levels. Our investigation provides initial evidence and information for the development of targeted therapies.

## Methods

### Patients and control subjects

The study population was composed of 203 healthy control subjects and 106 patients with non-small cell lung cancer (NSCLC) who had undergone curative surgical resection for primary lung cancer at Guangdong General Hospital. Tumor and matched normal lung specimens of each patient were stored in the hospital tumor bank. Samples were collected after obtaining informed consent. The project was approved by the ethics committee of Guangdong General Hospital (No: 200401).

The patient population had histologically confirmed NSCLC of all stages and consisted of 64 cases of adenocarcinoma, 30 cases of squamous cell carcinoma, five cases of adenosquamous carcinoma, and seven cases of large cell carcinoma. The median age of the patients was 60 years (range, 35 to 81 years). The controls were selected from a pool of healthy volunteers who had visited the hospital's check-up center. The median age of the controls was 43 years (range, 20 to 83 years).

### Genotyping

The genomic DNA of each control volunteer was isolated from whole-blood sample lymphocytes, using a Universal Genomic DNA Extraction kit (TaKaRa, Dalian, China). DNA from the normal tissue specimens of the lung cancer patients was extracted using Trizol reagent (Invitrogen, Life Technologies, Carlsbad, CA), according to the manufacturer's instructions. The frozen normal lung tissue of the patients obtained during surgical resection represented lung tissue located more than 5 cm from tumor tissue.

The sequences of the -271 G>A polymorphism of the KDR gene were assayed using PCR and DNA sequencing methods. The region was amplified using high-fidelity PrimerSTAR™ HS DNA polymerase (TaKaRa) and the following PCR primers designed according to the GenBank sequence (Accession No. NM_002253.1): forward, 5'-AGCTCCCACCCTGCACTGA-3 and reverse, 5'-CTGCCTTCCTCCTCCAGAG-3'. The product size was 414 bp, representing the region from -316 to 98 (relative to the transcription site) of the gene. The 50-μL PCR reaction contained 10 μL of 5× PCR buffer (Mg^2+ ^plus), 4 μL of dNTP (each 2.5 μM), 1.0 μL of forward and reverse primers (10 μM), 0.5 μL of DNA polymerase (25 U/μL), and 2.5 μL of DNA template, added water to a final 50 μL volume. The PCR conditions were 94°C for 2 min, followed by 35 cycles of 98°C for 10 s, 67°C for 15 s, and 72°C for 1 min, with a final extension at 72°C for 5 min. All PCR products were examined by 1% agarose gel electrophoresis and were purification from the gel. A BigDye Terminator v3.1 cycle sequencing kit (Applied Biosystems, Foster City, CA) and an ABI PRISM 310 Genetic Analyzer (Applied Biosystems) were used for sequencing.

### Real-Time PCR

The primer and probe design, total RNA isolation, cDNA synthesis, and quantification standards for real-time PCR were as described previously [12]. Briefly, total RNA was isolated from 50–100 mg of tissue, using Trizol reagent (Invitrogen) according to the manufacturer's instructions. The integrity of the total RNA was examined by 1% agarose gel electrophoresis, the quantity was determined based on absorbance at 260 nm (A260), and the purity was analyzed based on the absorbance ratio at 260 and 280 nm (A260/280) (Amersham Biosciences GeneQuant, Pittsburgh, PA). The cDNA was synthesized from 1 μg of total RNA using AMV reverse transcriptase XL (TaKaRa). β-actin served as an internal control. The lengths of the amplified PCR products were within a range of 50 to 150 bp, as recommended by Applied Biosystems for TaqMan assays. The probe sequence was designed to span exon borders of the gene, to avoid amplification of any contaminating genomic DNA. To create real-time PCR standards, KDR and β-actin were amplified using reverse transcriptase-PCR using specific primers. The amplicons were cloned into pMD18-T vector (TaKaRa) and confirmed by sequencing. The purified recombinant DNA was quantified and then serially diluted in ultra-pure water to final concentrations ranging from 10^7 ^to 10^1 ^copies/μL. For quantification standards, we used 1-μL aliquots of the ten-fold serial dilutions of plasmid DNA. A new standard curve was run for each real-time PCR, and each test run included a control containing no target DNA. Real-time PCR was performed independently at least two times, and the mean value was used for quantification. The amount of KDR mRNA was normalized to 10^6 ^copies of the β-actin internal control, and the data represent copy number/10^6 ^β-actin copies.

### Immunohistochemistry

We immunohistochemically examined the protein expression level of KDR in the frozen tumor specimens from 76 patients. The other 30 specimens could not be used for immunohistochemistry, because a reagent (RNAlater; Ambion, Austin, TX) had been previously added to the fresh specimens to stabilize and protect the RNA. For immunohistochemistry, 6- to 8-μm-thick frozen sections were cut, immediately fixed in cold methanol for 10 min, air-dried, and washed in PBS. Subsequently, endogenous peroxidase activity was blocked with 3% H_2_O_2 _for 10 min. The sections were incubated with ready-to-use anti-human KDR polyclonal antibody (Maixin-Bio, Fuzhou, China) overnight at 4°C and then rinsed with PBS. The bound primary antibody was detected using a ready-to-use secondary antibody kit (Histostain-Plus kit, Jingmei, Shenzhen, China) and the chromogenic substrate 3,3-diaminobenzidine tetrahydro-chloride. The specimens were counterstained with hematoxylin, mounted, and examined by light microscopy (Olympus BX50). Routine negative controls using PBS instead of the primary antibody were included to verify specificity.

All of the slides were reviewed concurrently by three of the authors (QX Lin, SJ An, and HJ Chen). KDR reactivity in tumor tissues was both nuclear and cytoplasmic. The staining intensity in both the cytoplasm and nuclei of tumor cells was scored on a 4-point scale: negative, 0; weak, 1; intermediate, 2; and strong, 3. Cells with a staining intensity score of zero were regarded as negative cells; those with scores of 1, 2, or 3 were regarded as positive. The percentage of positive cells was calculated by counting more than 1000 cancer cells in randomly selected high-power fields (10 × 40). Protein expression was considered positive when immunostaining was seen in at least 10% of the cancer cells. We identified two groups of patients based on the KDR level and location (cytoplasm or nucleus). In the negative group, both cytoplasmic and nuclear staining was negative; the positive group had either positive cytoplasmic or nuclear staining.

### Statistical analysis

Differences in genotype frequencies between groups were evaluated using Fisher's exact test, when appropriate. The correlation between different genotypes and KDR mRNA expression level was analyzed using one-way analysis of variance (ANOVA) or a *t*-test, as appropriate, with log transformation of the cDNA concentration data to fit a normal distribution. A two-tailed P value of < 0.05 was considered statistically significant.

## Results

### Frequency of the -271 G>A polymorphism genotypes and their relationships to clinical parameters and risk for lung cancer

**Figure 1:**
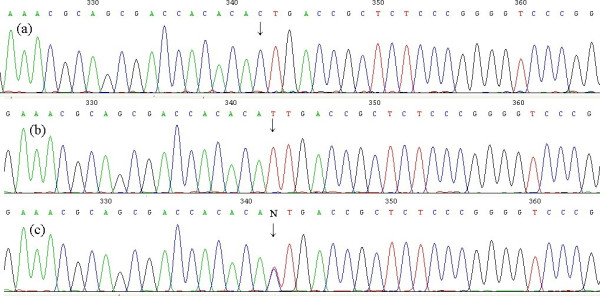
**Sequence graphs of the -271 G>A polymorphism of the KDR gene (sequenced using reverse primer)**. The arrays show the polymorphism site. (a) GG (CC) genotype; (b) AA (TT) genotype; (c) AG (TC) genotype.

The amplified products from all 106 specimens were successfully sequenced. A comparison of the sequences of 20 specimens with that of GenBank Accession No. NM_002253.1, using the Blast 2 program http://www.ncbi.nlm.nih.gov/blast/bl2seq/wblast2.cgi, did not reveal any mutations or polymorphisms in the investigated region except the -271 G>A polymorphism in the 5' UTR. Consequently, we focused on the -271 G>A polymorphism in all of the specimens and analyzed the frequency of its genotypes (Fig. 1). A search for -271 G>A polymorphisms in the single nucleotide polymorphism (SNP) bank of the National Center for Biotechnology Information (NCBI) yielded SNP rs7667298.

The frequencies of the AA, AG, and GG genotypes of the -271 G>A polymorphism in the NSCLC patients were 20.8% (22/106), 41.5% (44/106), and 37.7% (40/106), respectively. According to the NCBI SNP data bank http://www.ncbi.nlm.nih.gov/SNP/snp_ref.cgi?rs=7667298, the frequencies of the AA (TT) genotype of the -271 G>A polymorphism in European, Asian, and Sub-Saharan African populations were 17.6%, 4.5–4.7%, and 40.7%, respectively.

The frequencies of the AA and AG/GG genotypes in our healthy control subjects were 15.3% (31/203) and 84.7% (172/203), respectively. The genotype frequencies did not differ significantly between the lung cancer and control groups (χ^2 ^= 1.269, P = 0.264, Fisher's exact test).

The correlations between the -271 G>A polymorphism genotype and the patients' clinical parameters were analyzed using Fisher's exact test (Table 1). The distribution of genotypes was not related to gender, age, smoking status, tumor histology, or clinical stage of the patients (P > 0.05).

**Table 1:** Correlations between the genotypes of the -271 G>A polymorphism and clinical parameters of patients

Clinical Parameter	AA (%)	AG/GG (%)	χ^2^	P-value (two-sided)
Gender			0.058	0.811
Male	13 (20.0%)	52 (80.0%)		
Female	9 (22.0%)	32 (78.0%)		
Age, years			3.021	0.097
<60	14 (28.0%)	36 (72.0%)		
≥60	8 (14.3%)	48 (85.7%)		
Smoking status			3.664	0.086
Non-smoking	17 (27.0%)	46 (73.0%)		
Smoking	5 (11.6%)	38 (88.4%)		
Histology			0.123	0.810
Adenocarcinoma	14 (21.9%)	50 (78.1%)		
Others	8 (19.0%)	34 (81.0%)		
Stage			0.994	0.319
I/II	13 (18.1%)	59 (81.9%)		
III/IV	9 (26.5%)	25 (73.5%)		

Fisher's exact test, P (two-sided), N = 106

### The -271 G>A polymorphism affects the transcriptional level of the KDR gene, as detected by real-time PCR

In all, 103 specimens were available for mRNA analysis; the RNA of the other three specimens was degraded during the extraction process. ANOVA showed a marginally significant relationship between the KDR mRNA level and each of the three -271 G>A polymorphism genotypes, with mean values of 4.32, 4.08, and 4.03 for the AA, AG, and GG genotypes, respectively (F = 2.476, P = 0.089). When the AG and GG groups were combined, the AA genotype was significantly correlated with a higher KDR mRNA level in tumor tissues (mean, 4.32 ± 0.40), compared with the AG/GG genotype (mean, 4.06 ± 0.50, t = 2.178, P = 0.032, independent sample *t*-test, two-tailed; Table 2). Consequently, we combined the AG and GG groups (AG/GG group) for all subsequent analyses.

**Table 2:** Influence of genotypes of the -271 G>A polymorphism on KDR mRNA expression level

Genotype	N (%)	Mean ± SD
AA	21 (20.4%)	4.32 ± 0.40
AG/GG	82 (79.6%)	4.06 ± 0.50

Total	103 (100%)	4.11 ± 0.49

t = 2.718, P = 0.032 (two-tailed), independent samples *t*-test

### The -271 G>A polymorphism was not related to the KDR protein level

**Figure 2:**
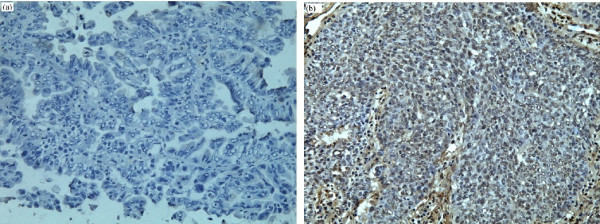
**KDR immunohistochemical staining of tumor tissues from lung cancer patients (original magnification, ×200)**. (a) Negative, (b) Positive.

Of the 76 tumor specimens analyzed immunohistochemically (Fig. 2), 14 (18.4%) were negative and 62 (81.6%) were positive for KDR protein expression. There was no correlation between the -271 G>A genotype and KDR protein level (Table 3).

**Table 3:** Association of -271 G>A polymorphism genotypes with KDR protein expression level

KDR protein expression	AA (%)	AG/GG (%)
Negative	3 (21.4)	11 (78.6)
Positive	15 (24.2)	47 (75.8)

χ^2 ^= 0.048, P = 1.000 (Fisher's exact test, two-sided).

## Discussion

Angiogenesis, which is the formation of new vessels from pre-existing vessels, is critical for the growth, maintenance, and metastasis of solid tumors. KDR, first isolated by Terman et al. [13], consists of an extracellular region with seven immunoglobulin-like domains, a transmembrane domain, and a tyrosine kinase domain. KDR is normally expressed in hematopoietic precursors as well as endothelial cells, nascent hematopoietic stem cells, and the umbilical cord stroma. However, KDR mRNA appears to be down-regulated in quiescent adult vasculature [14]. KDR activation occurs through ligand binding, which facilitates receptor dimerization and autophosphorylation of tyrosine residues in the cytoplasmic portion. The phosphotyrosine residues either enhance receptor catalytic activity or provide docking sites for downstream signaling proteins [15-21]. Hence, specific inhibitors of KDR tyrosine kinase are thought to be useful in treating cancer [22]. Some studies have shown that KDR is expressed strongly in the cytoplasm and nuclei of both cancer cells and peritumoral vessels [23-25]. Following stimulation with VEGF, KDR is translocated to the nucleus [26,27], demonstrating that VEGFR2/KDR is not a vasculature-restricted receptor but also has an additional role in cancer cell biology. This hypothesis is further supported by the frequent translocation of the receptor to the nuclei of cancer cells [23]. However, the autocrine VEGF/VEGFR system is not unique to malignant cells; it is also vital for the survival and growth of stem cells. The operation of autocrine pathways in stem cells indicates that these receptors are not always membrane-bound. Total KDR is reported to be widely distributed throughout the membrane, cytoplasm, and nucleus of a tumor cell [28].

In the present study, the frequency of the AA genotype of the -271 G>A polymorphism was higher among cancer patients than among controls (20.8% versus 15.3%), but the difference was not statistically significant (χ^2 ^= 1.269, P = 0.264, Fisher's exact test). These results suggest that the -271 G>A polymorphism may not be associated with a risk for lung cancer but may play roles in other steps of lung cancer development. Our study had the limitation of a sample size. A larger study with more than 1000 cancer cases and controls is needed to confirm our findings.

Compared with the AG/GG genotype, the AA genotype of the -271 G>A polymorphism was associated with a higher level of KDR mRNA expression in tumor tissues. This is the first suggestion that the -271 G>A polymorphism of the KDR gene may be functionally associated with the regulation of the gene's transcription level. A sequence in the 5' UTR of the gene contains the promoter region and multiple putative transcription-factor-binding sites [29]. We hypothesize that the -271 G>A polymorphism is contained in a response element of the gene and that the different KDR genotypes may affect the affinity of DNA binding protein for the gene promoter, resulting in different transcriptional activities. This hypothesis requires further investigation.

Although the -271 G>A polymorphism was related to the KDR mRNA level, it had no affect at the level of translation. This inconsistency between mRNA (transcription) and protein (translation) may be due to the complexity of the gene's expression or to multi-stage regulation mechanisms of the gene, which may be correlated with more efficient clearance of mRNA species (i.e., mRNA degradation). Proteins can be regulated at the levels of transcription and translation and may also be regulated post-translationally via the turnover rate. In addition, the apparent difference in regulation between KDR mRNA and protein expression might have resulted from differences in the methods of analysis. While the KDR mRNA was extracted from tumor tissues that included both tumor cells and extracellular matrix, the immunohistochemical assessment evaluated KDR protein only in tumor cells and not in the extracellular matrix.

## Conclusion

We analyzed variations in the expression levels of KDR mRNA and KDR protein in association with the KDR -271 G>A polymorphism genotype in tumor tissues of NSCLC patients. Ours is the first study to assess the relationship between the -271 G>A polymorphism of KDR and KDR mRNA and protein expression in NSCLC patients. Our results suggest that the -271 G>A polymorphism of the KDR gene may be a functional polymorphism associated with the regulation of KDR transcription. As KDR is a molecular target of cancer therapy, our clinical and KDR expression data may help to improve lung cancer therapy targeted to KDR. The functional polymorphism of KDR may be related to the response to or toxicity of targeted KDR therapy, which may provide further clues for tailoring individualized therapies.

## Abbreviations

KDR: kinase insert domain-containing receptor; NSCLC: non-small cell lung cancer; TKI: tyrosine kinase inhibitor.

## Competing interests

The authors declare that they have no competing interests.

## Authors' contributions

ASJ and WYL responsible for the research design, sample collection, processing, analysis, and writing the paper, CZH, LQX, SJ, CHJ, and LJY responsible for sample collection, processing, and analysis. The authors read and approved the final manuscript.

## Pre-publication history

The pre-publication history for this paper can be accessed here:

http://www.biomedcentral.com/1471-2407/9/144/prepub
